# Phonon Transport through Nanoscale Contact in Tip-Based Thermal Analysis of Nanomaterials

**DOI:** 10.3390/nano7080200

**Published:** 2017-07-28

**Authors:** Jay Dulhani, Bong Jae Lee

**Affiliations:** Department of Mechanical Engineering, Korea Advanced Institute of Science and Technology, Daejeon 34141, Korea; jay7591@gmail.com

**Keywords:** nanoscale constriction and contact, Boltzmann transport equation, phonon transport

## Abstract

Nanomaterials have been actively employed in various applications for energy and sustainability, such as biosensing, gas sensing, solar thermal energy conversion, passive radiative cooling, etc. Understanding thermal transports inside such nanomaterials is crucial for optimizing their performance for different applications. In order to probe the thermal transport inside nanomaterials or nanostructures, tip-based nanoscale thermometry has often been employed. It has been well known that phonon transport in nanometer scale is fundamentally different from that occurred in macroscale. Therefore, Fourier’s law that relies on the diffusion approximation is not ideally suitable for describing the phonon transport occurred in nanostructures and/or through nanoscale contact. In the present study, the gray Boltzmann transport equation (BTE) is numerically solved using finite volume method. Based on the gray BTE, phonon transport through the constriction formed by a probe itself as well as the nanoscale contact between the probe tip and the specimen is investigated. The interaction of a probe and a specimen (i.e., treated as a substrate) is explored qualitatively by analyzing the temperature variation in the tip-substrate configuration. Besides, each contribution of a probe tip, tip-substrate interface, and a substrate to the thermal resistance are analyzed for wide ranges of the constriction ratio of the probe.

## 1. Introduction

Recently, we are witnessing extreme miniaturization of thermal devices and other components [1,2,3]. Besides, rapid development in nanotechnology enables that nanostructures or nanomaterials have been widely employed in various applications for energy and sustainability, such as biosensing, gas sensing, solar thermal energy conversion, and passive radiative cooling [4,5,6,7]. Broad application of nanomaterials or nanostructures is posing new challenges for the thermal science community. One such challenge is in fundamental understanding of the geometry-induced effects on phonon transport in nanostructures and/or nanoscale contacts, which is crucial for optimizing the thermal performance of nanostructures.

Development and continuous evolution of a tip-based thermal microscopy are revolutionizing our perception about thermal transport at sub-micron scale. In a thermal microscope system, a nanometer-sized probe is often employed to interact with a specimen for measuring local temperature. Such tip-substrate configuration also has enormous practical applications, such as in data writing [8], chemical and biomolecular applications [9], nanofabrication [10] and nanoscale heat transfer analysis [11]. These applications can be broadly classified into two major area: nanomanufacturing and nanometrology. For the former application, the temperature of the probe is raised; thus, the probe acts as a localized heat source while interacting with a specimen (or a substrate). We are particularly interested in understanding the tip-substrate interaction in such systems.

It is known that the thermal conductivity of nanostructures is significantly lower than its bulk counterpart because of the phonon boundary scattering. Recent research also suggested that it is possible to alter the transport regime and thus the transport property by tailoring the geometry of nanostructures [12,13]. Besides, Jean et al. [14] concluded that the shape of the probe tip has a reasonable effect on probe’s measurement capacity. Thus, fundamental understanding of temperature distribution in the probe-substrate system is essential for the accurate use of tip-based thermal analysis.

Another area of our interest is in the quantification of thermal resistance for a tip-based thermal analysis system in which a probe with a nanoscale tip is in contact with a substrate. For macroscale thermal transport, the thermal contact resistance across the interface has been subjects of multiple studies [15,16]. As far as nanoscale thermal transport is concerned, the difference in the vibrational properties of phonon and partial transmission of phonon through the interface give rise to the thermal boundary resistance (TBR). Initial research has shown that thermal transport between the nanostructures can be limited by TBR [17]. Attempts are being made to model the thermal boundary resistance theoretically. Cooper et al. [15] derived the constriction resistance of a point contact between two semi-infinite objects of the same material using diffusion equation. This expression was strictly derived for the diffusive regime and will lead to significant error if transport is in quasi-ballistic regime. Taking it forward, Prasher [18] developed an analytical model to calculate the thermal resistance of nanoscale constrictions.

In light of the preceding discussion, the objective of present work is twofold: (i) fundamental understanding of phonon transport between a probe with nanoscale tip and a specimen (treated as a substrate made of the same material with the probe for simplicity) and (ii) quantification of thermal resistance of such geometry. These will be achieved by solving the phonon Boltzmann transport equation. In a recent study [13], the authors have used finite volume method to investigate the effect of constriction on phonon transport in thin films and nanowires. A similar approach is used here but for the different purpose.

## 2. Results and Discussion

Being aware of the complexity of the configuration under consideration, following assumptions are made. First, we assumed that perfect contact exists between the probe tip and the substrate; that is, there is no boundary scattering and/or resampling at the tip-substrate interface. Secondly, it is assumed that experiments occur in the vacuum; thus, convection loss through the air is absent.

**Figure 1:**
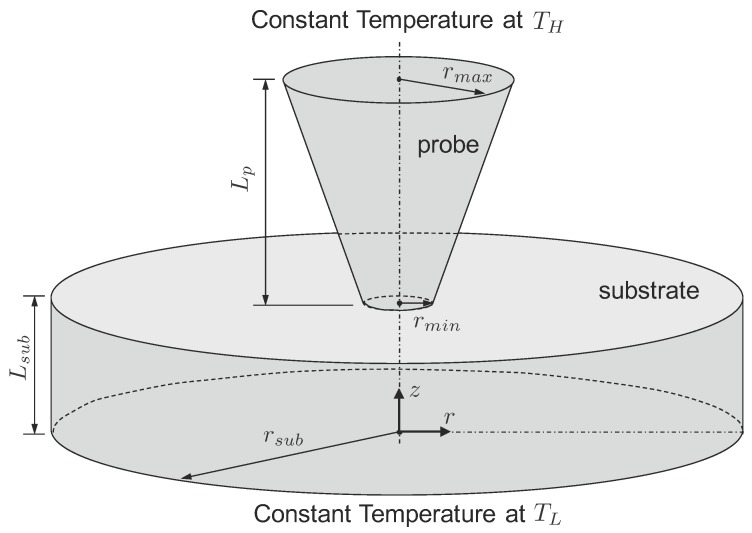
Geometric configuration of the probe tip and the substrate.

The schematic of geometric configuration considered in the present study is shown in Figure 1. Axisymmetric model is used here as it closely mimics the probe used in atomic force microscope. In Figure 1, length of the tip is $L_{p}$, and the radius of broad end of tip is $r_{max}$, whereas the radius of narrow end of tip is $r_{min}$. The length of substrate is $L_{sub}$ and the radius of substrate is $r_{sub}$. Broad end of the tip is maintained at temperature $T_{H}$, whereas, the bottom of the substrate is maintained at temperature $T_{L}$.

At steady state, the gray Boltzmann transport equation (BTE) can be written for an axisymmetric system with the relaxation time approximation as [19]:

(1) $$\frac{\mu }{r}\frac{\partial (re^{\prime \prime })}{\partial r}-\frac{1}{r}\frac{\partial (\eta e^{\prime \prime })}{\partial \phi }+\xi \frac{\partial e^{\prime \prime }}{\partial z}=\frac{e^{0}-e^{\prime \prime }}{\Lambda }$$

where $e^{\prime \prime }$ is the net phonon energy density, $e^{0}$ is the equilibrium energy density, μ, η, ξ are direction cosines, and Λ is the phonon mean free path. Details about formulation and boundary conditions can be found elsewhere [13,19,20,21]; thus, they are not repeated here.

All the results presented here are for Silicon at 300 K. Thermophysical properties required for the BTE simulations are calculated by phonon branch averaging procedure [22]. Silicon’s properties are group velocity $v_{g}=1804$ m/s, mean free path Λ=260.4 nm, and specific heat $c_{p}=0.93\times 10^{6}$ J/m${}^{3}\cdot$K. For all results presented here, angular domain of 2π (i.e., 0≤θ≤π and 0≤ϕ≤π due to symmetry) is discretized into 128×128 non-overlapping control angles. Further refinement in it does not bring any reasonable change to the computational accuracy. The geometric dimensions are $L_{sub}=3\Lambda$ and $r_{sub}=4\Lambda$ for the substrate (refer to Figure 1). As far as the probe tip is concerned, its length is fixed to be $L_{p}=1500$ nm and radius of the broad end varies as $r_{max}=100$, 150, or 200 nm. Radius of the narrow end (i.e., size of the tip-substrate contact) is determined according to the constriction ratio, $C_{r}=r_{min}/r_{max}$.

**Figure 2:**
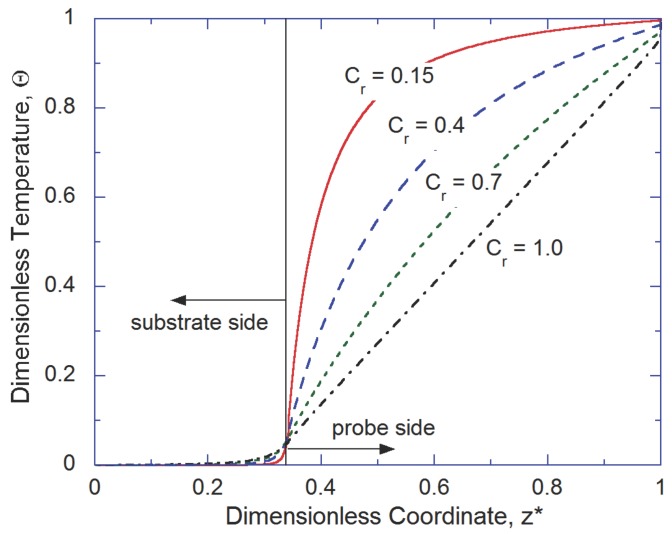
Temperature distribution along the *z*-axis when $r_{max}=100$ nm for various $C_{r}$ values.

Qualitative features of the tip-substrate interaction are explored first by analyzing the temperature distribution. Figure 2 represents the non-dimensional temperature distribution along the *z*-axis of the tip-substrate system when $r_{max}=100$ nm. In the figure, temperature is normalized as $\Theta =\left(T-T_{L}\right)/\left(T_{H}-T_{L}\right)$, and the *z*-axis is normalized as $z^{*}=z/\left(L_{p}+L_{sub}\right)$. For the case of $C_{r}=1$, a constant temperature gradient is observed on the probe side, which implies that the phonon transport is mainly in diffusive regime. Substrate’s response is similar to the case when the substrate is subjected to a localized surface heating, which is not surprising as in the considered configuration the tip-substrate interface acts as a localized heat source for the substrate. With increase in the degree of constriction (i.e., lower $C_{r}$ values), phonon transport in the probe side becomes increasingly ballistic due to the constriction effect (i.e., phonon boundary scattering), as evident from nearly constant temperature profile in the probe side for $C_{r}=0.15$ (i.e., $r_{min}=15$ nm). Moreover, irrespective of the constriction ratio, the temperature of the substrate is found to be roughly constant. This is due to high thermal resistance across the tip-substrate interface. Another interesting observation is a drastic change in the temperature gradient across the tip-substrate interface. Such trend points to the non-equilibrium nature of phonon transport across the nanoscale tip-substrate contact even though perfect contact is assumed.

**Figure 3:**
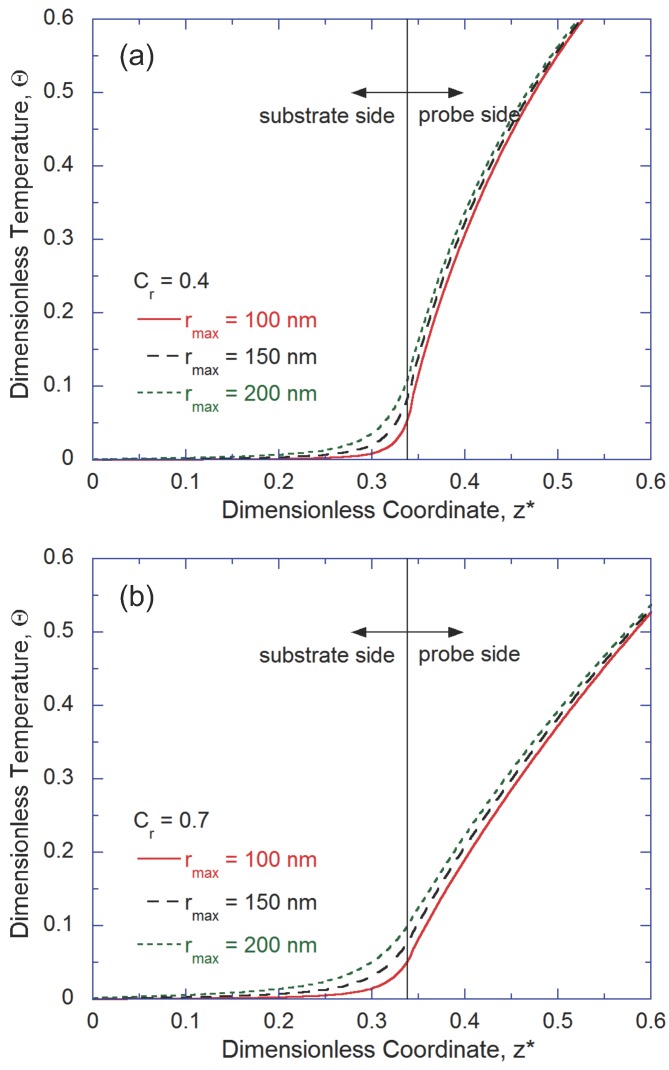
Temperature distribution along the *z*-axis for constant constriction ratio of (**a**) $C_{r}=0.4$ and (**b**) $C_{r}=0.7$, while varying $r_{max}$ values.

Next, we want to explore the effect of absolute dimensions on the qualitative features of thermal transport. To achieve this, constriction ratio of the probe tip is kept constant while the absolute dimension ($r_{max}$ and $r_{min}$) is varied. Figure 3 shows the temperature distribution along the *z*-axis of the tip-substrate system for different $r_{max}$ values. As can be seen from Figure 3, if $C_{r}$ is kept constant, variation in $r_{max}$ (or in $r_{min}$) does not affect the temperature distribution significantly. Thus, it would be inferred that for the range of length scale considered, the constriction ratio and not the absolute dimension is the critical parameter for determining thermal transport inside the tip-substrate system. Similar observations were also reported by Cheney [23]. It should be also noted that size confinement could alter the mean free path of the low-frequency phonons more than that of the high-frequency phonons [24]. To properly model the spectrally dependent phonon mean free path, the phonon dispersion relation as well as the three phonon scattering relaxation time should be considered, which is beyond the scope of this study.

The study of the thermal resistance is critical as it can significantly affect the phonon transport in nanostructures. The heat transfer rate *Q* from $T_{H}$ to $T_{L}$ is obtained directly from the BTE simulations. Various components of the thermal resistance are defined as: total thermal resistance of the tip-substrate system $R_{total}=\left(T_{H}-T_{L}\right)/Q$, thermal resistance in the substrate $R_{sub}=\Delta T_{sub}/Q$, thermal resistance at the interface between the tip and the substrate $R_{inter}=\Delta T_{inter}/Q$, and thermal resistance in the probe tip $R_{tip}=R_{total}-R_{sub}-R_{inter}$. Here, the temperature profile in the substrate is extrapolated linearly to calculate $\Delta T_{sub}$, and $\Delta T_{inter}$ is calculated by averaging the temperature of control volumes adjacent to the interface and then taking their difference. Care has been taken to ensure that the cell adjacent to the interface are at equal distance from the tip-substrate interface. The similar procedure was also used by Jean et al. [14].

**Figure 4:**
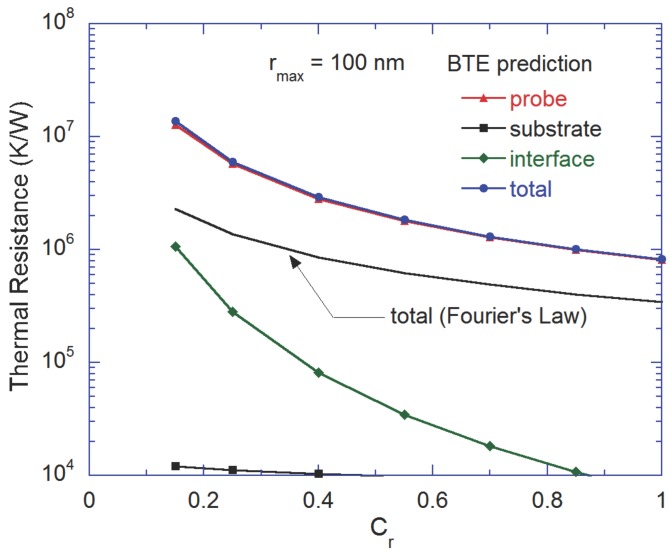
Variation of the thermal resistance with respect to the constriction ratio when $r_{max}=100$ nm.

Figure 4 shows variation of the thermal resistance with constriction ratio for $r_{max}=100$ nm. As we observed from the temperature profile, the effect of the substrate is broadly restricted by high thermal resistance at the interface. In fact, $R_{sub}$ is less than 2% of the total resistance and thus can be neglected. Besides, $\left(R_{tip}+R_{inter}\right)$ is much higher than the total resistance predicted by Fourier’s law, and this difference increases as $C_{r}$ decreases (i.e., the degree of constriction increases). Fourier’s law is found to substantially underpredict the thermal resistance as compared to that predicted by the BTE. In Figure 4, $R_{tip}$ and $R_{inter}$ increase with increase in the degree of constriction. Interestingly, when observed from non-dimensional basis, $C_{r}$ has more profound impact on $R_{inter}$ than on $R_{tip}$. Please note that in reality, the probe tip cannot make a perfect contact with the substrate. In other words, there must be additional boundary scattering at the tip-substrate interface due to lattice mismatch. Therefore, $R_{inter}$ estimated in this work indicates the minimum possible value.

**Figure 5:**
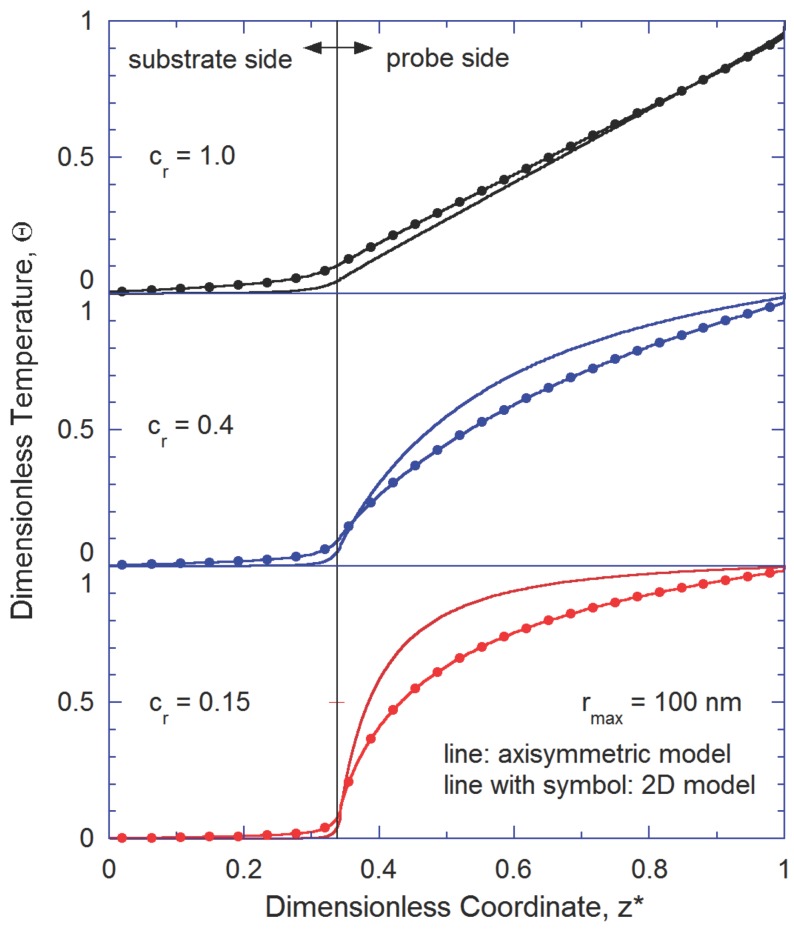
Temperature distribution along the *z*-axis as predicted by the axisymmetric model and the 2-D model when $r_{max}=100$ nm. In the 2-D model, the width of broad end of tip is $2\times r_{max}$, whereas the width of narrow end of tip is $2\times r_{min}$.

In general, phonon boundary scattering depends on the surface area to volume ratio. In this regard, a two-dimensional (2-D) model of the BTE may differently predict when compared with the axisymmetric model. Here, thermal transport in the tip-substrate system has been analyzed using both the axisymmetric model and the 2-D model. The Gray BTE code utilized for the 2-D model is same as used previously by the authors [13]. Figure 5 shows the temperature distribution along the *z*-axis of the tip-substrate system for $r_{max}=100$ nm. The qualitative comparison indicates that constriction can play a dominating role in governing the thermal transport especially when the degree of constriction is high (i.e., when $C_{r}$ value is low). For a given heat flux, the thermal resistance of different components is proportional to the temperature drop across the corresponding component. It can be noticed that the interface provides significantly higher thermal resistance to phonons in the axisymmetric model. A possible reason behind such trend is that the area to volume ratio is greater for the axisymmetric case.

## 3. Summary

In summary, we have investigated the phonon transport through the constriction formed by a probe itself as well as the nanoscale contact between the probe tip and the specimen based on the gray Boltzmann transport equation. We observed that constriction of the probe tip could play a dominating role in governing the phonon transport especially when the degree of constriction is high. It was also found that the combined thermal resistance of the probe tip and the tip-substrate interface predicted by the BTE is much greater than the total resistance predicted by Fourier’s law and this difference increases as the degree of constriction increases. Lastly, the tip-substrate interface is found to offer significantly higher resistance to phonons in the axisymmetric model as compared to the 2-D model. The insights obtained from the present study may be applied to improve our understanding of a tip-based device.
